# Genome-Wide Association Study to Identify Genetic Factors Linked to HBV Reactivation Following Liver Transplantation in HBV-Infected Patients

**DOI:** 10.3390/ijms26010259

**Published:** 2024-12-30

**Authors:** Joonhong Park, Dong Yun Kim, Heon Yung Gee, Hee Chul Yu, Jae Do Yang, Shin Hwang, YoungRok Choi, Jae Geun Lee, Jinsoo Rhu, Donglak Choi, Young Kyoung You, Je Ho Ryu, Yang Won Nah, Bong-Wan Kim, Dong-Sik Kim, Jai Young Cho, The Korean Organ Transplantation Registry (KOTRY) Study Group

**Affiliations:** 1 Department of Laboratory Medicine, Jeonbuk National University College of Medicine and Hospital, Jeonju 54907, Republic of Korea; miziro@jbnu.ac.kr; 2 Research Institute of Clinical Medicine of Jeonbuk National University-Biomedical Research Institute of Jeonbuk National University Hospital, Jeonju 54907, Republic of Korea; yjd@jbnu.ac.kr; 3 Department of Internal Medicine, Yonsei University College of Medicine, Seoul 03722, Republic of Korea; jlkyy@yuhs.ac; 4 Department of Pharmacology, Graduate School of Medical Science, Brain Korea 21 Project, Yonsei University College of Medicine, Seoul 03722, Republic of Korea; 5 Department of Surgery, Jeonbuk National University College of Medicine and Hospital, Jeonju 54907, Republic of Korea; 6 Department of Surgery, Asan Medical Center, College of Medicine University of Ulsan, Seoul 05505, Republic of Korea; shwang@amc.seoul.kr; 7 Department of Surgery, Seoul National University Hospital, Seoul National University College of Medicine, Seoul 03080, Republic of Korea; choiyoungrok@gmail.com; 8 Department of Surgery, The Research Institute for Transplantation, Yonsei University College of Medicine, Seoul 03722, Republic of Korea; drjg1@yuhs.ac; 9 Department of Surgery, Samsung Medical Center, Sungkyunkwan University School of Medicine, Seoul 06351, Republic of Korea; jinsoo.rhu@samsung.com; 10 Department of Surgery, Daegu Catholic University School of Medicine, Daegu 42472, Republic of Korea; dnchoi@cu.ac.kr; 11 Department of Surgery, College of Medicine, The Catholic University of Korea, Seoul 06591, Republic of Korea; yky602@catholic.ac.kr; 12 Department of Surgery, Pusan National University Yangsan Hospital, Pusan National University School of Medicine, Yangsan 50612, Republic of Korea; ryujhhim@hanmail.net; 13 Department of Surgery, Ulsan University Hospital, University of Ulsan College of Medicine, Ulsan 44033, Republic of Korea; nahyw@uuh.ulsan.kr; 14 Department of Hepato-Biliary-Pancreatic Surgery, Ajou University School of Medicine, Suwon 16499, Republic of Korea; drbwkim@ajou.ac.kr; 15 Department of Surgery, Korea University College of Medicine, Seoul 02841, Republic of Korea; kimds1@korea.ac.kr; 16 Department of Surgery, Seoul National University Bundang Hospital, Seoul National University College of Medicine, Seongnam 13620, Republic of Korea; jychogs@gmail.com

**Keywords:** hepatocellular carcinoma, HBV reactivation, liver transplantation, hepatitis B, genome-wide association study, *RGL1*, *CDCA7L*, *AQP9*

## Abstract

This study utilized a genome-wide association study (GWAS) to investigate the genetic variations linked to the risk of hepatitis B virus (HBV) reactivation in patients who have undergone liver transplantation (LT), aiming to enhance understanding and improve clinical outcomes. Genotyping performed on a selected patients from the Korean Organ Transplantation Registry (KOTRY) data using high-throughput platforms with the Axiom Korea Biobank array 1.1. The discovery cohort included 21 patients who experienced HBV reactivation (cases) and 888 patients without HBV reactivation (controls) following LT. The replication cohort consisted of 5 patients with HBV reactivation (cases) and 312 patients without HBV reactivation (controls) after LT. Additive logistic regression analysis was conducted using PLINK software ver 1.9, with adjustments for age and gender. The GWAS findings from the discovery cohort were validated using the replication cohort. The GWAS identified several single-nucleotide polymorphisms (SNPs) in the *RGL1*, *CDCA7L*, and *AQP9* genes that were significantly linked to HBV reactivation after LT, with genome-wide significance thresholds set at *p* < 10^−7^. Down-regulation of *RGL1* cDNAs was observed in primary duck hepatocytes infected with duck HBV. Overexpression of *CDCA7L* was found to promote hepatocellular carcinoma cell proliferation and colony formation, whereas knocking down CDCA7L inhibited these processes. Additionally, the absence of AQP9 triggered immune and inflammatory responses, leading to mild and scattered liver cell pyroptosis, accompanied by compensatory liver cell proliferation. This study provides critical insights into the genetic factors influencing HBV reactivation after LT, identifying significant associations with SNPs in *RGL1*, *CDCA7L*, and *AQP9*. These findings hold promise for developing predictive biomarkers and personalized management strategies to improve outcomes for HBV-infected LT recipients.

## 1. Introduction

Chronic hepatitis B virus (HBV) infection is a pervasive global health concern, affecting an estimated 248 million individuals worldwide in 2010. Of those infected, approximately 15–40% face the risk of severe liver complications, resulting in 600,000 to 1.2 million deaths annually [1]. The Republic of Korea is classified as a region with intermediate HBV endemicity, with an estimated prevalence of 3%, as reported in the 2016 Korea National Health and Nutrition Examination Survey. Chronic HBV infection is a leading contributor to liver cirrhosis and hepatocellular carcinoma (HCC) [2]. However, advancements in HBV vaccination, nationwide screening initiatives, and antiviral treatments have significantly improved management outcomes [3]. Most liver transplant recipients with HBV-related liver disease achieve favorable post-transplant outcomes under prophylactic therapy, with minimal risks of HBV recurrence or graft failure. Over the last ten years, second-generation nucleos(t)ide analogues (NA) therapy have revolutionized long-term viral suppression, effectively controlling HBV both pre- and post-transplant. Consequently, some transplant centers are reassessing the need for indefinite use of expensive prophylactic treatments. However, achieving hepatitis B surface antigen (HBsAg) seroconversion in chronic HBV patients or recovering from acute hepatitis does not necessarily confer immunity against reactivation. Occult hepatitis B infection (OBI), characterized by undetectable HBsAg but measurable HBV DNA in the serum or liver, represents an additional concern [4]. HBV reactivation, especially during immunosuppressive treatments or after liver transplantation (LT), can result in severe complications, including graft failure and mortality [5]. Furthermore, the potential for HBV transmission from HBsAg-negative but anti-hepatitis B core (HBc)-positive donors to HBV-naïve recipients highlights the importance of comprehensive donor and recipient screening protocols [6]. Notably, recipients of livers from anti-HBc-positive donors face an approximately 78% risk of de novo HBV infection compared to just 0.5% when the donor is anti-HBc-negative [7]. To reduce the likelihood of OBI in transplant scenarios, it is essential to evaluate serum HBV DNA in anti-HBc-positive donors and recipients while considering other clinical factors [8]. The persistent shortage of organs for LT has led to a growing reliance on marginal donors, including those with hepatic steatosis or positive viral hepatitis serologies. Historically, anti-HBc-positive donor status was considered a contraindication for transplantation in HBV-naïve recipients, primarily due to the absence of routine prophylactic measures like lamivudine or hepatitis B immunoglobulin (HBIG) at the time [6]. Currently, a combination of HBIG and antiviral therapies has been implemented to reduce the risk of HBV transmission effectively. Despite these advancements, the use of grafts from anti-HBc-positive donors continues to be a topic of debate in regions with a high prevalence of HBV. Denying the use of such grafts could significantly reduce the already limited availability of suitable donor organs, posing a dilemma in transplantation practices [6].

Genetic variations in the host may play a role in determining the risk of HBV reactivation following LT. To identify genes associated with susceptibility to HBV-related conditions and transplant outcomes, two key strategies are typically employed. The first approach involves genome-wide association studies (GWAS) [9], a prominent method in genetic research for complex diseases, including HBV infection. In the initial stages of GWAS, patient samples are examined to analyze a wide array of candidate single nucleotide polymorphism (SNP) loci using advanced technologies such as TaqMan probes, SNPstream genotyping, SNaPshot genotyping, SNP chips, and others. These SNPs then undergo rigorous validation in multiple stages to establish their significance. The second approach focuses on traditional candidate gene selection, where specific genes are chosen based on prior theoretical knowledge or empirical evidence indicating their involvement in HBV-related conditions or therapeutic responses. This method involves analyzing SNP genotype frequencies in patient and control groups to verify their potential association. Notably, SNPs in the cytotoxic T lymphocyte antigen-4 (CTLA-4) +49 and CD86 +1057 loci have been identified as influential in LT outcomes, particularly in the context of allograft acceptance [10,11]. For instance, the G/G genotype of CTLA-4 +49 has been associated with a reduced risk of HBV recurrence in Chinese LT recipients [11]. CD86 and CTLA-4 perform contrasting roles in T-cell activation, with CD86 promoting activation, while CTLA-4 inhibits it.

Understanding insights into the genetic mechanisms that drive HBV reactivation is essential for formulating effective predictive tools and management strategies. This study utilizes a GWAS to investigate the genetic variations linked to the risk of HBV reactivation in patients who have undergone LT, aiming to enhance understanding and improve clinical outcomes.

## 2. Results

Genotyping performed on a selected patients from the Korean Organ Transplantation Registry (KOTRY) data using high-throughput platforms with the Axiom Korea Biobank array 1.1. As a result, the GWAS identified 7.2 million SNPs that met imputation and quality control standards within the discovery cohort. A genomic inflation factor (λ) of 1 confirmed the absence of systemic bias related to population substructure. This finding was further validated by the distribution of *p*-values on the quantile–quantile plot. The discovery cohort of the GWAS involved 909 participants, comprising 21 cases and 888 controls, and identified a single locus achieving genome-wide significance (*p* < 5 × 10^−8^). The most significant SNP, with a *p*-value of 4.32 × 10^−9^, was mapped to the *RGL1* gene on chromosome 1q25.3. Three variants associated with this locus were subsequently tested in an independent replication cohort. In the replication GWAS, which included 317 participants (5 cases and 312 controls), no genome-wide significant signals were detected. However, the three *RGL1* variants linked to HBV reactivation in the discovery cohort demonstrated marginal significance (*p* = 0.06) and consistent directional effects in the replication cohort (Table 1).

**Figure 1:**
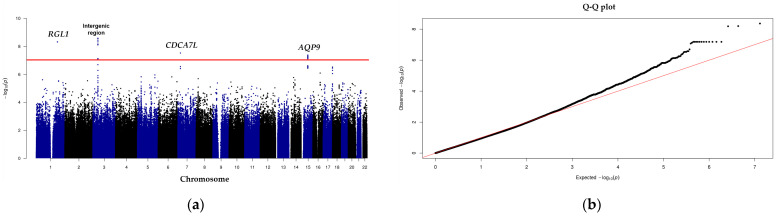
Genome-wide association study (GWAS) revealed that *RGL1* is associated with HBV reactivation after LT. (**a**) Manhattan plot of GWAS of HBV reactivation in Korean liver transplantation recipient. Variants are plotted on the *x*-axis of the Manhattan plot according to their chromosomal position, and their –log10 (*p*-value) is shown on the *y*-axis. The red line shows the genome-wide significance threshold (*p* < 5 × 10^−8^). (**b**) QQ-plot of *p*-values from a GWAS comparing patients with HBV reactivation after LT to those in a control group in the entire cohort. The red line indicates the expected distribution under the null hypothesis in the Q-Q plot.

In the entire cohort, GWAS identified multiple SNPs in the *RGL1*, *CDCA7L*, and *AQP9* genes that were significantly associated with HBV reactivation following LT, meeting the genome-wide significance threshold (*p* < 10^−7^). A regional association plot highlighting the loci for *RGL1*, *CDCA7L*, and *AQP9* is displayed in Figure 1. Previous research has demonstrated that *RGL1* expression is down-regulated in primary duck hepatocytes infected with duck HBV. Furthermore, overexpression of *CDCA7L* has been shown to enhance HCC cell proliferation and colony formation, while its suppression has the opposite effect, inhibiting these processes. Similarly, AQP9 deficiency has been linked to immune and inflammatory responses that lead to scattered liver cell pyroptosis, coupled with compensatory liver cell proliferation.

## 3. Discussion

HBV recurrence can arise from either the immediate reinfection of the liver graft by circulating HBV particles, delayed activation of extrahepatic HBV reservoirs such as peripheral blood mononuclear cells (PBMCs), or a combination of both mechanisms [12]. Despite advancements in transplantation medicine, HBV recurrence remains a critical challenge affecting the prognosis of patients with HBV-related end-stage liver disease undergoing LT. Several GWASs have explored the connections between genetic variations and HBV recurrence following LT [13,14,15,16,17]. For instance, the variant rs11536889 genotype in the *TLR4* gene has been significantly associated with a lower risk of HBV recurrence posttransplant (*p* = 0.040, odds ratio = 0.390, 95% confidence interval: 0.159–0.957), suggesting that polymorphisms in the 3′ untranslated regions of *TLR4* may provide a protective effect in Han Chinese LT recipients [14]. Another study identified the *IL-28B* gene rs8099917 genotype as correlating with elevated alanine aminotransferase (ALT) and aspartate aminotransferase (AST) levels in HBV-related LT recipients (*n* = 140). Recipients carrying the G allele (GG + GT) exhibited significantly higher ALT and AST levels (*p* < 0.05), although no direct relationship was found between *IL-18* and *IL-28B* polymorphisms and HBV recurrence in recipients or donors. The presence of IFN-γ was identified as a protective factor against HBV recurrence, while the rs8099917 G allele was linked to increased hepatocyte injury, suggesting potential benefits from more aggressive antiviral therapy for individuals with this allele [15]. In addition, the *IL-15* rs10519613 polymorphism in LT recipients was linked to a higher risk of HCC recurrence. Those with the CA/AA genotypes of this polymorphism exhibited poorer disease-free and overall survival rates compared to individuals with the CC genotype, positioning this genetic variant as a potential biomarker for predicting clinical outcomes in HCC patients undergoing LT [16]. Furthermore, donor liver mutations in rs1979277 (G > A) were significantly associated with an elevated risk of HBV recurrence after transplantation (*p* = 0.042). Survival analysis in HCC patients revealed that mutations in donor liver SNPs rs1801133 (G > A) and rs1979277 (G > A) were significant risk factors for HBV recurrence (*p* < 0.05). However, none of the studied SNPs showed an association with HBV recurrence in non-HCC recipients (*n* = 97, *p* > 0.05). Genetic variations in the donor liver genes, particularly within the one-carbon metabolism pathway, were also identified as key factors influencing post-transplant HBV recurrence and adversely affecting post-transplant survival [17].

In our KOTRY cohort analysis, GWAS revealed several SNPs within these genes that were strongly linked to HBV reactivation following LT, reaffirming their importance. The results of this study emphasize the significance of *RGL1*, *CDCA7L*, and *AQP9* in the reactivation of HBV. These genes demonstrated significant associations with HBV reactivation, reaching genome-wide significance thresholds (*p* < 10^−7^) across the study cohorts. *RGL1* plays a pivotal component of the RAS signaling pathway, which regulates essential cellular functions such as proliferation, differentiation, and transformation [18]. Alterations in the RAS pathway are well-documented contributors to various cancers [19]. The functional activities of RAS are mediated through its interactions with downstream signaling proteins, known as RAS effectors, which activate a cascade of signal transduction events. Proteins from the RAL guanine nucleotide dissociation stimulator family, as downstream effectors, play critical roles in the RAS and RAL signaling pathways [20]. *RGL1* operates within a RAS-mediated signaling pathway distinct from the RAF pathway, transmitting RAS signals to activate *CFOS* gene expression and RAL [21]. The widespread expression of *RGL1* across human tissues underlines its essential role in RAS signaling. In animal studies, specific gene expressions, including *RGL1*, were altered during duck HBV infection in primary duck hepatocytes, where *RGL1* was notably down-regulated [22]. This pattern of modulation suggests parallels in HBV pathogenesis between human HBV and duck HBV, members of the same hepadnavirus family [23].

*CDCA7L*, a member of the JPO protein family, has been identified as a target gene of the c-Myc oncogene [24,25]. Its counterpart, *CDCA7*, exhibits high expression during the blast crisis phase of chronic myelogenous leukemia, and transgenic mice overexpressing *CDCA7* are at a significantly increased risk of developing lymphoid malignancies. These findings underscore *CDCA7*’s pivotal role in the progression of hematologic cancers [26]. Similarly, *CDCA7L* is overexpressed in several cancers, where it promotes cellular proliferation, particularly in medulloblastoma cells. Both *CDCA7* and *CDCA7L* interact with c-Myc, amplifying its oncogenic potential and contributing to tumorigenesis. The elevated expression of *CDCA7L* in medulloblastoma cells and its ability to enhance c-Myc-mediated transformation further implicate it in neoplastic processes, highlighting its role in cancer progression [27]. Tian et al. demonstrated that *CDCA7L* is markedly up-regulated in HCC [28]. In HCC cells, *CDCA7L* facilitates the progression of the cell cycle from the G0/G1 phase to the S phase. Overexpression of *CDCA7L* was found to enhance cell proliferation, colony formation, soft agar colony formation, and tumorigenicity in SK-hep-1 and Focus HCC cell lines. In vivo studies using nude mouse models have shown that *CDCA7L* overexpression significantly accelerates tumor growth. Conversely, silencing *CDCA7L* expression suppressed cell proliferation and colony formation and significantly reduced tumor burden in YY-8103 and MHCC-97H HCC cells. This effect is mediated through activation of the ERK1/2 signaling pathway and regulation of cell cycle progression [28].

*AQP9*, a member of the aquaglyceroporin subgroup within the aquaporin protein family, facilitates the transport of water and glycerol, playing a critical role in hepatic gluconeogenesis, especially during starvation. This protein exhibits broad solute permeability, enabling hepatocytes to transport molecules such as glycerol and urea, although it does not facilitate the movement of beta-hydroxybutyrate [29,30,31]. Its expression is regulated by nutritional status and circulating insulin levels, highlighting its key role in metabolic processes [32]. Found predominantly in the plasma membranes of hepatocytes, AQP9 is integral to liver function due to its solute transport capabilities [33]. AQP9 has also been linked to tumor progression, particularly in HCC. Studies suggest that it may act as an inhibitor of HCC progression through pathways such as Wnt/β-catenin signaling [34], epithelial-to-mesenchymal transition [35], and the reactive oxygen species (ROS)-HIF-1α signaling cascade [36]. Despite its potential significance, the exact molecular mechanisms underlying AQP9’s role in immune infiltration, reduced expression in HCC, and its diagnostic and prognostic implications remain unclear. Lower levels of AQP9 in HCC have been associated with poor clinical outcomes, possibly through its involvement in immune response regulation and key signaling pathways. This highlights AQP9 as a promising target for immunotherapy in liver cancer. Moreover, with the increasing prevalence of non-alcoholic fatty liver disease, AQP9 is gaining attention as a therapeutic target in HCC due to its function as a water and glycerol channel [37]. Experimental studies in murine models have further revealed the metabolic consequences of AQP9 deficiency. Disruption of glycerol metabolism in AQP9-deficient mice led to hepatocyte death, accompanied by inflammatory cell infiltration and metabolic imbalances that caused reduced body weight. AQP9 knockout was also associated with immune and inflammatory responses, including mild, scattered pyroptosis of liver cells followed by compensatory liver cell proliferation. These processes culminated in hepatocyte regeneration, emphasizing AQP9’s role in maintaining immune homeostasis and liver function under normal conditions. Its dual involvement in immune regulation and metabolism underscores its potential as a therapeutic target for both liver diseases and HCC [38].

In LT for HCC, the dynamics of HBV reinfection differ markedly from those observed in benign liver diseases due to additional complexities introduced by tumor immunology and anti-tumor treatments. In HCC patients, polymorphisms in rs1979277 (*SHMT1*) and rs1801133 (*MTHFR*) have been identified as significant risk factors for HBV recurrence. The *MTHFR* gene, located on chromosome 1 at 1p36.6, encodes methylenetetrahydrofolate reductase, a vital enzyme involved in folate and one-carbon metabolism. This enzyme converts 5,10-methylenetetrahydrofolate into 5-methyltetrahydrofolate, the primary circulating form of folate [39]. Mutations in the rs1801133 and rs1801131 loci lead to reduced activity of the MTHFR enzyme [40]. Specifically, rs1801133 has been associated with an increased susceptibility to various viral infections, including human papillomavirus types 16 and 18 [41], as well as HBV infection [42]. In comparison, the clinical impact of rs1801131 appears to be less pronounced. Unlike rs1801133, rs1801131 mutations have a limited effect on serum homocysteine levels, as suggested by certain studies [43].

OBI and HBV variants associated with severe liver diseases often persist in LT recipients despite the administration of prophylactic treatments. Achieving complete virological eradication of HBV in post-LT patients remains challenging, as low-level or occult HBV infection can persist in plasma and PBMCs, even with potent prophylaxis. HBV covalently closed circular DNA (cccDNA) is frequently detected in the PBMC compartment, emphasizing the virus’s resilience. Moreover, HBV genotypes and variants commonly linked to cirrhosis and HCC are frequently present in post-LT patients and can dominate the quasi-species population. Although immunosuppression does not necessarily prevent HBV control or the silencing of cccDNA transcription, it fails to achieve complete virological clearance in LT recipients with HBV-related end-stage liver disease [44]. The diversity of HBV across different compartments corresponds with prior findings, which indicate that treatment-resistant HBV variants occur at varying frequencies in plasma, liver tissue, and PBMCs [45]. Notably, variants associated with HCC and cirrhosis—such as T1753V, A1762T, and G1764A—are detected at high frequencies in both the plasma and PBMCs of post-LT patients. Kim et al. reported an elevated risk of HCC in patients harboring persistent low-level HBV despite receiving NA therapy [46]. The continued presence of HBV, including oncogenic variants, in LT recipients underscores that virological risk factors linked to severe or end-stage liver disease can persist even with HBV prophylaxis. This highlights the need for more effective strategies to manage HBV and mitigate long-term risks in these patients [44].

Risk genes contributing to HBV reactivation have been identified through GWAS and candidate gene analyses. These genes often play roles in immune regulation, influencing the host’s response to HBV. For instance, variations in human leukocyte antigen (HLA) alleles have been associated with chronic hepatitis B susceptibility, highlighting their impact on antigen presentation and immune response modulation [47]. Additionally, cytokine signaling genes, such as interleukin-10 (IL-10) and interferon-gamma (IFN-γ), are crucial in modulating immune responses, potentially affecting HBV reactivation risk. Certain genes may also influence viral replication pathways by encoding host factors that interact with viral proteins or DNA. The HBV X protein (HBx) is known to interact with various cellular proteins, affecting viral replication and contributing to hepatocarcinogenesis [48]. Furthermore, risk genes associated with cellular stress and apoptosis can regulate stress responses or programmed cell death, impacting the balance between viral persistence and reactivation. HBV infection has been shown to induce oxidative stress, leading to DNA damage and apoptosis, which may influence viral reactivation [49]. Second, the reactivation of HBV is a complex process influenced by various genetic factors that affect viral replication and the host immune response. cccDNA serves as a stable template for HBV transcription and is central to viral persistence. Its activity can be modulated by chromatin remodeling and epigenetic modifications. For instance, interferon-α (IFN-α) has been shown to repress viral transcription through epigenetic mechanisms involving chromatin remodeling complexes [50]. Proteins such as histone acetyltransferases and deacetylases regulate cccDNA activity by modifying its chromatin structure. Epigenetic repression of cccDNA can be enhanced by treatments combining IFN-α and small-interfering RNA, leading to reduced viral transcription [51]. Genetic variations in the HLA region can influence immune recognition of HBV-infected cells, affecting the balance between immune tolerance and antiviral responses. Specific HLA alleles have been associated with different outcomes in viral infections, including HBV [52]. Variants in interferon lambda genes, such as *IFNL3* (formerly *IL28B*), can impact the efficacy of the interferon response against viral infections. Certain polymorphisms are associated with impaired clearance of viruses like hepatitis C, suggesting a potential role in HBV reactivation during immune suppression [53]. HBV proteins, including HBx, can increase intracellular ROS, leading to oxidative DNA damage. The virus may exploit host DNA repair mechanisms to maintain cccDNA stability, and variations in genes involved in these pathways could influence susceptibility to HBV reactivation [54]. Third, the impact of genetic risk factors on HBV reactivation varies with clinical and therapeutic contexts, particularly during immunosuppressive treatments for cancer, organ transplantation, or autoimmune conditions. Patients undergoing immunosuppressive therapies, such as immune checkpoint inhibitors (e.g., PD-1 and CTLA-4 inhibitors), are at increased risk of HBV reactivation. A systematic review highlighted that immune checkpoint inhibitors are associated with a high risk of HBV reactivation in HBsAg-positive patients [55]. The interplay between host genetics and viral characteristics, including HBV genotypes and specific mutations, influences reactivation risk. Mutations in the HBV genome, such as those in the pre-core region, can lead to variants that do not produce HBeAg, complicating treatment and increasing the risk of prolonged infection and liver cirrhosis [56]. The American Gastroenterological Association recommends screening for HBV in patients at moderate or high risk who will undergo immunosuppressive drug therapy, emphasizing the importance of identifying and managing potential reactivation risks [57].

This study has several limitations. The discovery cohort included only 21 cases and 888 controls, while the replication cohort consisted of 5 cases and 312 controls. Such small sample sizes diminish statistical power and restrict the broader applicability of the findings. The marginal significance noted in the replication cohort highlights this limitation, as limited sample sizes increase the likelihood of false-positive or false-negative results and reduce the ability to identify less common genetic variations linked to HBV reactivation. Furthermore, we explored public GWAS databases containing data on HBV recurrence after LT in HBsAg-positive hepatitis B patients, recruited in other ethnic populations, to validate the SNP markers located on *RGL1*, *CDCA7L*, and *AQP9* genes identified in the entire cohort. However, the relevant data were not available. The analysis included a large number of SNPs despite the relatively small number of cases, heightening the risk of overfitting. This may result in associations that fail to replicate in larger or more diverse populations. Additionally, the study was conducted exclusively on a Korean population, restricting its relevance to other ethnic or genetic groups. Variations in genetic predispositions and environmental influences across different populations could lead to differing risks of HBV reactivation, making the findings less generalizable beyond this demographic. While SNPs in *RGL1*, *CDCA7L*, and *AQP9* were linked to HBV reactivation, the study lacks detailed experimental validation to confirm their functional roles in the disease process. Without such validation, the causal connections between these genes and HBV reactivation remain speculative, limiting the translational applicability of the results. Although the analysis accounted for age and gender, other important confounding variables—such as immunosuppressive therapy protocols, donor characteristics, and coexisting medical conditions—were not considered. These factors could independently or synergistically influence HBV reactivation, potentially confounding the observed associations. Before the introduction of effective antiviral therapies and immunoprophylaxis, the recurrence rate of HBV after LT ranged from 80% to 100%. However, with the perioperative and early post-transplant use of HBIG and the administration of NAs such as tenofovir or entecavir, which have low resistance profiles, the recurrence rate has significantly decreased to less than 5–10% in well-managed cases. Thus, the low rate of HBV recurrence after LT in HBV-infected patients, largely due to effective prophylactic therapy, is expected to have a meaningful impact. Clinicians consider combination therapy with high-dose HBIG and high-genetic-barrier NAs to be the most effective prophylaxis regimen; however, its high cost continues to limit broader implementation [58,59]. Furthermore, the study concentrated solely on SNPs and did not investigate other genetic variations, such as copy number variations, insertions, deletions, or structural variants, which may also contribute to HBV reactivation risk. This narrow focus could have excluded key genetic contributors. Environmental and lifestyle factors, including diet, alcohol use, and exposure to hepatotoxic substances, were also not evaluated. The study did not explore epigenetic mechanisms such as DNA methylation or histone modification, which may significantly affect gene expression relevant to HBV reactivation. While certain genetic associations were identified, the underlying biological pathways and mechanisms leading to HBV reactivation remain inadequately understood. This gap limits the ability to develop targeted therapeutic or preventive strategies. Moreover, the KOTRY database, despite being a comprehensive resource, may introduce biases related to data collection, reporting practices, or participant selection, potentially affecting the generalizability of the findings to broader clinical contexts.

These limitations underscore the necessity for further research to better understand the broader implications of these findings. Future studies should involve larger, multicenter cohorts to enhance statistical power and ensure the findings are generalizable across diverse populations. Including participants from various ethnic and geographical backgrounds will provide a more comprehensive understanding of genetic variations influencing HBV reactivation. Highlighting the need for such investigations in the discussion could significantly enhance the study’s impact on the field. Additionally, both in vitro and in vivo functional experiments are essential to confirm the biological roles of the identified genes. A thorough analysis of potential confounders, including a broader range of clinical and environmental variables, is crucial to minimize bias and refine the understanding of HBV reactivation risk. Integrating epigenetic and longitudinal data will allow researchers to explore dynamic changes, such as DNA methylation or histone modifications, that may influence reactivation over time. Finally, the adoption of multi-omics approaches—including transcriptomics, proteomics, and metabolomics—could complement GWAS findings by providing a more holistic perspective on the molecular mechanisms underlying HBV reactivation. Such integrative methodologies will not only validate current findings but also pave the way for novel therapeutic and preventive strategies.

## 4. Materials and Methods

### 4.1. Study Subjects

The study population was sourced from KOTRY, a large-scale, nationwide, multicenter cohort established to prospectively gather data related to transplantation across the Republic of Korea [60]. Numerous leading national hospitals and transplantation centers contributed to the KOTRY initiative. Patient enrollment occurred consecutively following transplantation procedures, with subsequent follow-up spanning from January 2014 to December 2019. The registry encompassed extensive patient information, including demographic characteristics, comorbidities, laboratory findings, immunosuppressive therapy protocols (induction and maintenance), and other clinically significant outcomes. Blood samples from 2218 patients were stored for genotyping and analyzed using the KOTRY database. For this study, the inclusion criteria were patients aged 18 years or older, seropositivity for HBsAg at the time of transplantation, and a post-transplant follow-up duration of at least two years. Exclusion criteria encompassed co-infection with hepatitis C virus, death within the first year after transplantation, and cases of re-transplantation. All patients included in the study were eligible for post-transplant HBV prophylaxis. Post-transplant HBV prophylaxis was administered following the established protocols of Asan Medical Center [58]. Patients with less than one year of follow-up, incomplete medical or laboratory records, or other exclusion criteria were omitted. Consequently, 1226 patients were ultimately included in the study. The discovery cohort consisted of 21 individuals experiencing HBV reactivation (cases) and 888 without reactivation (controls) post liver transplantation. Meanwhile, the replication cohort included 5 cases of HBV reactivation and 312 controls without reactivation. The demographics and clinical characteristics of HBV-infected patients who received liver transplantation are summarized in Table 2.

### 4.2. Genotyping, Imputation, and Quality Control in the GWAS

Genotype data were obtained using the Korean Chip (K-CHIP), developed by the K-CHIP consortium in collaboration with the Center for Genome Science under the Korea National Institute of Health (4845–301, 3000–3031). Genomic DNA was extracted from whole blood samples treated with EDTA, with 200 ng of DNA processed for genotyping using the Axiom Korea Biobank array 1.1 (Affymetrix, Santa Clara, CA, USA). Genotype imputation was conducted utilizing HapMap phase II NCBI GRCh37/hg19 genetic maps with SHAPEIT2 ver 2.20. Genome-wide imputation followed, employing the cosmopolitan reference panel from phase III of the 1000 Genomes Project and performed with IMPUTE2 ver 2.3.2. Stringent quality control criteria were applied to the discovery samples, excluding SNPs with a total call rate below 97%, a minor allele frequency of less than 1%, or a Hardy–Weinberg equilibrium *p*-value below 1.0 × 10^−3^. Additionally, imputed variants with an imputation information score (r^2^ info) below 0.9 were filtered out using QCTOOL ver 1.4, and the final data were converted into PLINK format via GTOOL ver 0.7.5. After quality control, 7,227,238 autosomal SNPs were included in the GWAS for the discovery cohort, while 7,227,963 autosomal SNPs were used in the replication cohort.

### 4.3. Statistical Analysis

Genome-wide associations between HBV reactivation following LT and imputed genetic variants were analyzed using PLINK ver 1.9 (Free Software Foundation Inc., Boston, MA, USA) [61], with adjustments made for age and sex. Logistic regression was employed to evaluate these associations under an additive genetic model, utilizing both Python and PLINK for the analysis. The λ was calculated based on median chi-square statistics to assess potential population stratification or systemic bias. Visualization of the results included a Manhattan plot and a quantile–quantile plot, both generated using R software ver 4.3.2 (http://cran.r-project.org/; accessed on 31 October 2023). Additionally, regional association plots were created with LocusZoom [62]. All statistical analyses were conducted with IBM SPSS Statistics software version 24.0 (IBM Inc., Armonk, NY, USA).

## 5. Conclusions

This study provides critical insights into the genetic factors influencing HBV reactivation after LT, identifying significant associations with SNPs in *RGL1*, *CDCA7L*, and *AQP9*. These findings hold promise for developing predictive biomarkers and personalized management strategies to improve outcomes for HBV-infected LT recipients. By elucidating the genetic underpinnings of HBV reactivation, the study contributes to advancing personalized medicine in liver transplantation. However, the findings emphasize the need for further research to validate these genetic markers in larger, ethnically diverse populations. Experimental studies are essential to uncover the functional mechanisms of *RGL1*, *CDCA7L*, and *AQP9* in HBV reactivation. Integrating environmental, epigenetic, and multi-omics data will provide a holistic understanding of how genetic and non-genetic factors interact to influence reactivation risks. Addressing these gaps will enhance the translational potential of these findings, paving the way for targeted therapeutic interventions and improved post-transplant care.

## Figures and Tables

**Table 1:** Summary of significant SNPs for HBV reactivation after liver transplantation with *p* < 5 × 10^−8^ in a Korean population.

Chromosome	Position	rs Number	Closest Gene ^a^	Allele	MAF	OR ^b^ (Dis)	*p* ^c^ (Dis)	OR ^b^ (Rep)	*p* ^c^ (Rep)
1q25.3	183743399	rs147360905	*RGL1*	C > T	0.013	21.92 (7.82–61.44)	4.32 × 10^−9^	9.71 (0.89–105.75)	0.062
	183659995	rs139293187	*RGL1*	C > T	0.013	20.96 (7.50–58.52)	6.34 × 10^−9^	9.71 (0.89–105.75)	0.062
	183600161	rs144590902	*RGL1*	T > C	0.013	20.96 (7.49–58.43)	6.54 × 10^−9^	9.71 (0.89–105.75)	0.062

^a^ The closest gene was annotated using the UCSC Genome Browser (GRCh37/hg19). ^b^ Odds ratio and 95% confidence interval (CI) of minor allele. ^c^ *p*-values were determined by logistic regression analysis using an additive model. Abbreviations: MAF, minor allele frequency; OR, odds ratio; Dis, discovery cohort; Rep, replication cohort.

**Table 2:** Demographics and Clinical Characteristics of HBV-Infected Patients Who Received Liver Transplantation.

	Entire Cohort (*n* = 1226)	Discovery Cohort (*n* = 909)	Replication Cohort (*n* = 317)
HBV reactivation, n (%)	26 (2)	21 (2)	5 (2)
No HBV reactivation, n (%)	1200 (98)	888 (98)	312 (98)
Male, n (%)	994 (81)	736 (81)	258 (81)
Age, year (range)	55 (23–76)	54 (23–74)	56 (29–76)
Donor type			
Living, related, n (%)	1001 (82)	745 (82)	256 (81)
Living, unrelated, n (%)	206 (17)	148 (16)	58 (18)
Dual graft, n (%)	19 (2)	16 (2)	3 (1)
Donor serology			
HBsAg-negative, n (%)	1225 (100)	908 (100)	317 (100)
Anti-HBs-positive, n (%)	862 (70)	627 (69)	235 (74)
Anti-HBc-negative, n (%)	929 (76)	691 (76)	238 (75)
Recipient serology			
HBsAg-positive, n (%)	1129 (92)	843 (93)	286 (90)
Anti-HBs-negative, n (%)	1124 (92)	842 (93)	282 (89)
Anti-HBc-positive, n (%)	1162 (95)	865 (95)	297 (94)
Post-transplant HBV prophylaxis			
None, n (%)	9 (1)	4 (0)	5 (2)
HBIG only, n (%)	383 (31)	316 (35)	67 (21)
Antiviral therapy only, n (%)	10 (1)	2 (0)	8 (3)
HBIG + antiviral therapy, n (%)	824 (67)	587 (65)	237 (75)

HBV, hepatitis B virus; HBsAg, hepatitis B surface antigen; Anti-HBs, hepatitis B surface antibody; Anti-HBc, hepatitis B core antibody; HBIG, hepatitis B immunoglobulin.

## Data Availability

The data used in this study are available from the KOTRY office upon reasonable request.
